# RACK1 promotes cancer progression by increasing the M2/M1 macrophage ratio via the NF‐κB pathway in oral squamous cell carcinoma

**DOI:** 10.1002/1878-0261.12644

**Published:** 2020-02-20

**Authors:** Hongxia Dan, Sai Liu, Jiajia Liu, Dongjuan Liu, Fengying Yin, Zihao Wei, Jiongke Wang, Yu Zhou, Lu Jiang, Ning Ji, Xin Zeng, Jing Li, Qianming Chen

**Affiliations:** ^1^ State Key Laboratory of Oral Diseases National Clinical Research Center for Oral Diseases Chinese Academy of Medical Sciences Research Unit of Oral Carcinogenesis and Management West China Hospital of Stomatology Sichuan University Chengdu China; ^2^ Department of Oral Pathology Department of Dental Materials School of Stomatology China Medical University Shenyang China

**Keywords:** macrophage polarization, NF‐κB, oral squamous cell carcinoma, RACK1, tumor‐associated macrophages

## Abstract

Receptor for activated C kinase 1 (RACK1) has been shown to promote oral squamous cell carcinoma (OSCC) progression, and RACK1 expression levels have been negatively correlated with prognosis in patients with OSCC. Here, we investigated the impact of RACK1 OSCC expression on the recruitment and differentiation of tumor‐associated macrophages. High RACK1 expression in OSCC cells correlated with increased M2 macrophage infiltration in tumor samples from a clinical cohort study. Moreover, the combination of RACK1 expression and the M2/M1 ratio could successfully predict prognosis in OSCC. OSCC cells with high RACK1 expression inhibited the migration of THP‐1 cells, promoted M2‐like macrophage polarization *in vitro*, and increased the proportion of M2‐like macrophages in a xenograft mouse model. Moreover, both M1‐ and M2‐like macrophage polarization‐associated proteins were induced in macrophages cocultured with RACK1‐silenced cell supernatant. A mechanistic study revealed that the expression and secretion of C‐C motif chemokine 2 (CCL2), C‐C motif chemokine 5 (CCL5), interleukin‐6 (IL‐6), and interleukin‐1 (IL‐1) are closely related to RACK1 expression. In addition, blocking nuclear factor‐kappa B (NF‐κB) could promote M2‐like macrophage polarization. These results indicate that RACK1 and the M2/M1 ratio are predictors of a poor prognosis in OSCC. RACK1 promotes M2‐like polarization by regulating NF‐κB and could be used as a potential therapeutic target for antitumor immunity.

## Graphical abstract

High receptor for activated C kinase 1 expression OSCC cells could inhibit the expression and secretion of proinflammatory factors and macrophage chemokines by regulating nuclear factor‐kappa B, thus inhibiting the massive recruitment of macrophages and promoting M2‐like macrophage polarization, inducing a chronic smoldering inflammation microenvironment and promoting the development of tumors.

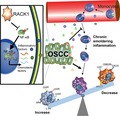

**Abbreviations**
IHC, immunohistochemical
IL, interleukin
M0: total macrophages
M1: classically activated macrophages
M2: alternatively activated macrophages
NF‐κB: nuclear factor‐kappa B
OSCC: oral squamous cell carcinoma
PMA, phorbol‐12‐myristate‐13 acetate
RACK1: receptor for activated C kinase 1
TAM: tumor‐associated macrophages
TCGA, The Cancer Genome Atlas

## 1 Introduction

Oral squamous cell carcinoma (OSCC) is one of the most common human cancers (Torre et al., 2015) affecting more than 400 000 people every year. Due to its high recurrence and metastasis rates, OSCC has a high mortality rate and a poor prognosis. In recent decades, despite the enormous progress in diagnosis and treatments such as radiotherapy and chemotherapy, the 5‐year survival rate is no more than 50% (Panzarella *et al.*, 2014). A better understanding of the mechanisms underlying the occurrence and development of OSCC will facilitate the development of novel treatment options.

According to their different functions, macrophages fall into two categories: classically activated macrophages (M1) for killing tumor cells, and alternatively activated macrophages (M2) for promoting tumor cells (Brown *et al.*, 2017). Tumor‐associated macrophages (TAM) are macrophages present in high numbers in the tumor microenvironment (TME) (Sica *et al.*, 2008). It is well acknowledged that TAM mainly have an M2‐like phenotype. Clinical and experimental evidence has shown that TAM promote the development and progression of most tumor types (Ruffell and Coussens, 2015; Tan *et al.*, 2018b). There are many macrophages in OSCC tissues, and TAM are also believed to participate in OSCC progression (Kubota and Moriyama, 2017; Petruzzi *et al.*, 2017; Sun *et al.*, 2018). The most commonly used markers for M1 macrophages are CD11c, CD80 and HLA‐DR. CD206, CD163 and CD204 are useful for M2 detection. Moreover, CD68 is considered a pan‐macrophage marker (Guo *et al.*, 2018; Gustafson *et al.*, 2015; Han *et al.*, 2016; Motomura *et al.*, 2015; Olesch *et al.*, 2015; Salmi *et al.*, 2018; Tan‐Garcia *et al.*, 2017; Wang *et al.*, 2014a).

Receptor for activated C kinase 1 (RACK1), a highly conserved WD40 repeat scaffold protein, is a multifaceted signaling adaptor. RACK1 has been confirmed to take part in multiple biological events, including cell migration(Li *et al.*, 2012b), virus infection (Majzoub *et al.*, 2014), neural development (Wehner *et al.*, 2011; Xu *et al.*, 2015), angiogenesis (Berns *et al.*, 2000; Zhou *et al.*, 2014) and cancer metastasis (Li *et al.*, 2012a). In our previous study, we found that RACK1, an organ‐specific prognostic predictor in OSCC, could promote the malignant biological behavior of OSCC (Liu *et al.*, 2018b; Zhang *et al.*, 2016). However, the effect of RACK1 on the tumor immunological microenvironment in OSCCs is poorly understood. Whether RACK1 is related to the recruitment and differentiation of TAM and the underlying mechanisms are unclear.

Here, we showed that RACK1 can suppress the activation of nuclear factor‐kappa B (NF‐κB), regulate the expression of IL‐6, CCL5 and CSF secreted by tumor cells, inhibit the massive recruitment of macrophages and severe inflammatory reactions, induce a chronic smoldering inflammatory microenvironment and promote the development of tumors. RACK1 could serve as a potential target in antitumor immunity against OSCC.

## 2 Materials and methods

### 2.1 Patients and follow‐up

One cohort included 45 patients with OSCC who underwent surgery between 2005 and 2009. All patients were informed of sample collection and usage. The tissue samples were collected and used in accordance with a protocol approved by the Human Research Ethics Committees of the West China Hospital of Stomatology, Sichuan University and Guangdong Provincial Stomatological Hospital. This research was performed in accordance with the Declaration of Helsinki and according to national and international guidelines. All animal studies were approved by the Animal Care and Use Committee, State Key Laboratory of Oral Diseases, in compliance with the Guide for the U.S. Public Health Service's policy on the humane care and use of laboratory animals. Animals were housed in compliance with the Association for the Assessment and Accreditation of Laboratory Animal Care International guidelines. The other cohort (TCGA), comprising 460 patients with HNSCC, was obtained from the TCGA database.

### 2.2 Immunohistochemical (IHC) assay and analysis

The IHC assay was performed as previously described (Liu *et al.*, 2018a). Briefly, sections were incubated overnight at 4 °C with a RACK1 antibody (Santa Cruz Biotechnology, Santa Cruz, CA, USA, RACK1: sc‐17754, 1 : 200 dilution), CD11b antibody (Abcam, Cambridge, MA, USA, anti‐CD11b antibody, ab133357, 1 : 4000 dilution), CD206 antibody (Abcam, anti‐mannose receptor antibody, ab64693, 1 : 5000 dilution) and CD68 antibody (Abcam, anti‐CD68 antibody ab955, 1 : 200 dilution) after antigen retrieval. The cells were then detected with a ChemMate DAKO EnVision Detection Kit (DAKO, Copenhagen, Denmark). Finally, the sections were counterstained with Mayer's hematoxylin. The staining was assessed by three independent investigators without any knowledge of the clinico‐pathological data.

The following criteria were used to score the RACK1 staining: staining intensity: 0 – no detectable staining, 1 – light yellow, 2 – medium yellow, 3 – deep yellow or 4 – brown; and staining proportion: 1 (≤ 10%), 2 (10–50%), 3 (50–80%) or 4 (≥ 80%). The product of the two scores was considered the final score (nine levels: 1, 2, 3, 4, 6, 8, 9, 12 and 16). For TAM, the scoring method was as follows: in the software graphics processing function of imagescope (Vista, CA, USA), the scope of the tumor stroma was framed and the percentage of positive cells within the frame calculated. The value calculated by the software was used for the patient survival analysis. The above score was divided into nine categories to match the final RACK1 score and to conveniently perform a correlation analysis between the two indicators.

### 2.3 Reagents

Phorbol‐12‐myristate‐13 acetate (PMA) and the NF‐κB inhibitor BAY11‐7082 were obtained from Beyotime (Shanghai, CHN). Primary antibodies were from Santa Cruz Biotechnology [CREB (sc‐271); p‐CREB (10E9) (sc‐81486); STAT3 (sc‐8019); PPARγ (sc‐7273); p‐PPARγ S112 (sc‐28001); GAPDH (sc‐47724); Actin (sc‐8432)], Abcam [RACK1 (ab129084); STAT1 (ab92506); p‐STAT1 (S727) (ab109461); CD206 (ab64693)], Cell Signaling Technology (Danvers, MA, USA) [c‐Jun (#9165); NF‐κB p65 (#8242); p‐NF‐κB p65 (#3033); p‐mTOR (Ser2448) (#5536); p‐ERK (Thr202/Tyr204) (#4370)]. Antibodies used for flow cytometry, including anti‐human CCR7‐FITC (#353216) and CD206‐PE (#321106), and anti‐mouse CD11b‐APC (#301310), F4/80‐FITC (#123107) and CD206‐PE (#141705) were purchased from BioLegend (San Diego, CA, USA).

### 2.4 Cell culture

Cal‐27 cells were obtained from the American Type Culture Collection (Manassas, VA, USA). HSC‐3 and HSC‐4 cells were purchased from the Japanese Collection of Research Bioresources (JCRB, Shinjuku, Japan). RAW264.7 cells were purchased from the Chinese Academy of Sciences (ATCC Number: TIB‐71, Beijing, China). THP‐1 cells were the kind gift of X. Zhou (State Key Laboratory of Sichuan University). Cal‐27, HSC‐3, HSC‐4 and RAW264.7 cells were maintained in Dulbecco's modified Eagle's medium (DMEM) containing 10% FBS. THP‐1 monocytes were cultured in RPMI medium supplemented with 10% FBS. The generation of stable HSC‐3 cells with low RACK1 expression (sh‐RACK1) and HSC‐4 cells with RACK1 overexpression (OE‐RACK1) was performed as previously described (Zhang *et al.*, 2016). All cells were cultured under a humidified atmosphere with 5% CO_2_ at 37 °C.

### 2.5 Cell migration assay

The supernatants from infected HSC‐3 (sh‐NC, sh‐RACK1, OE‐vector or OE‐RACK1) cells cultured in serum‐free DMEM were harvested after 48 h. The infected HSC‐3 cell supernatants were filtered using 0.45‐μm polyvinylidene difluoride membrane filters and concentrated by ultrafiltration (Amicon Ultra 3K; Merck Millipore, Billerica, MA, USA). The Bradford method was employed to determine the protein concentrations. THP‐1 cells (2 × 10^7^ per well) or RAW264.7 cells (1 × 10^6^ per well) were added to the upper compartment of a 24‐well Transwell chamber and then cocultured for 24 h with DMEM containing 40 μg·mL^−1^ infected OSCC supernatants. The lower compartment contained DMEM supplemented with 2% FBS. The migrated THP‐1 cells were counted by flow cytometry, and the migrated RAW264.7 cells were fixed, stained with Cell Stain Solution (Sigma‐Aldrich, St Louis, MO, USA) and photographed under a light microscope.

### 2.6 Cell invasion assay

THP‐1 cells (2 × 10^7^ per well) or RAW264.7 cells (1 × 10^6^ per well) were added to the upper compartment of a 24‐well Transwell chamber coated with Matrigel and cocultured for 48 h with DMEM containing 40 μg·mL^−1^ infected OSCC supernatants. The lower compartment contained DMEM supplemented with 2% FBS. The numbers of invaded THP‐1 cells were counted by flow cytometry, and the invaded RAW264.7 cells were fixed, stained with Cell Stain Solution and photographed under a light microscope.

### 2.7 Western blot analysis

Cell protein was extracted with RIPA lysis buffer. The protein expression levels of RACK1, STAT3, c‐Jun, NF‐κB, p‐NF‐κB, STAT1, p‐STAT1, p‐ERK, PPARγ, CREB, p‐CREB, p‐mTOR and actin in OSCC cells and macrophages were examined.

### 2.8 Analysis of human cytokines and genes

HSC‐3 cells grown to 70% confluence in a 10‐cm dish were lipofected with 100 nm si‐RACK1 or si‐NC using Lipofectamine 2000. The cells and supernatants were harvested after 48 h. The variations in human angiogenesis genes and proteins in si‐RACK1 and si‐NC cells and supernatants were detected by a Human Cytokine Array Panel A (ARY005; R&D Systems, Minneapolis, MN, USA) and a whole human gene expression profile PCR array (KangChen Bio‐tech, Shanghai, China).

### 2.9 Flow cytometry analysis

THP‐1 cells were adjusted to 1 × 10^6^ cells per mL in RPMI medium with 100 ng·mL^−1^ PMA for 24 h to induce THP‐1 differentiation into macrophages. The cells were then cocultured with 40 μg·mL^−1^ infected OSCC supernatants for 8 h. PE‐, APC‐ and FITC‐conjugated anti‐human CCR7 and CD206 and anti‐mouse F4/80 and CD11b antibodies were used to analyze the surface antigen expression of macrophages and homogeneous isotypes were used as controls. The cells were washed and then stained for 30 min at 4 °C. The stained cells were analyzed by flow cytometry (EXL™; Beckman Coulter, Brea, CA, USA). Data analysis was performed using flowjo software (Tree Star, Ashland, OR, USA).

### 2.10 Tumor xenograft model

Female BALB/c nude mice (4–6 weeks of age) were used and assigned randomly to two groups (five mice in each group): sh‐NC and sh‐RACK1. A human OSCC tumor model was established by subcutaneously injecting 1 × 10^6^ cells (0.1 mL) into the right upper flanks of the mice. From the fresh tumor tissues, single cells were isolated using collagenase IV and then stained with CD206, CD11b and F4/80 antibodies for 30 min at 4 °C. The stained cells were washed and resuspended in PBS/0.1% bovine serum albumin plus azide. Flow cytometry was performed and the results were analyzed by flowjo software.

### 2.11 Neutralization

The supernatant from sh‐NC cells was harvested after 48 h of culture in serum‐free DMEM with the NF‐κB inhibitor BAY 11‐7082 (0.1%, 10 μm in DMSO; Beyotime) or 0.1% DMSO. The methods used for protein concentration and measurement for the supernatants were the same as those described in the cell migration assay. The method used to analyze macrophage surface antigen expression was the same as that described for flow cytometry analysis.

### 2.12 Statistical analysis

Overall survival (OS) was estimated using the Kaplan–Meier method with a log‐rank test for the univariate analysis. A two‐sided *P*‐value < 0.05 was considered significant. The results are expressed as the mean ± SD of at least three different experiments.

## 3 Results

### 3.1 High levels of RACK1 expression are associated with high numbers of tumor‐infiltrating M2 macrophages and a poor prognosis in OSCC

**Figure 1:**
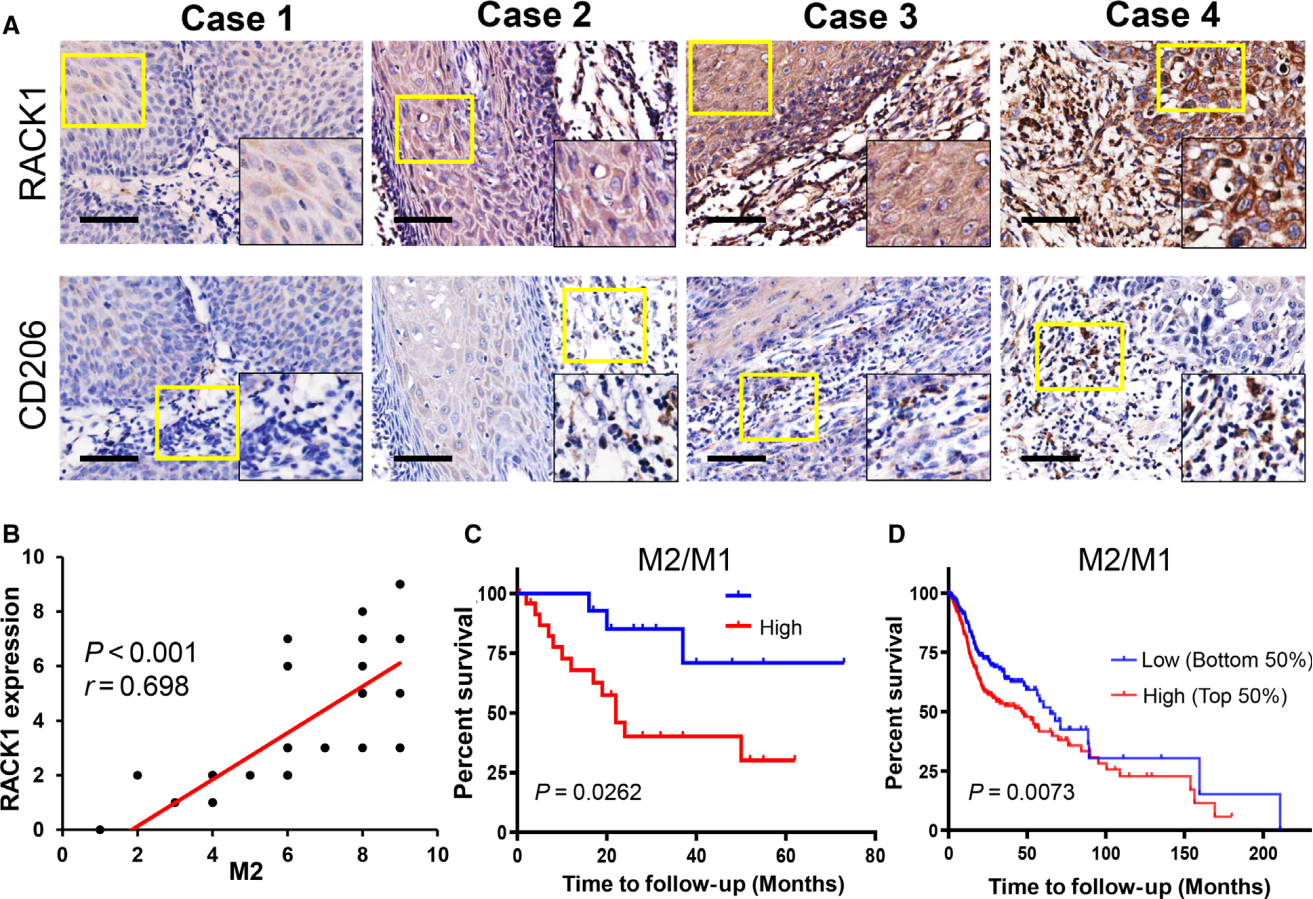
The M2/M1 ratio is positively correlated with the RACK level and is associated with a poor prognosis in OSCC. (A) IHC staining of 36 paraffin‐embedded OSCC sections with human antibodies against RACK1 and CD206 (scale bar: 100 μm). (B) Statistical analysis revealed that the expression intensity of RACK was positively correlated with the number of M2 macrophages (*r* = 0.698, *P* < 0.001). (C) Kaplan–Meier survival analysis revealed that a high M2/M1 ratio indicated a poor OS in 36 OSCC patients (*P* = 0.0262). (D) TCGA database analysis showed that a high M2/M1 ratio indicated a poor OS in human primary oral cancers (*P* < 0.01).

According to previous studies, an increased level of RACK1 indicates a poor clinical outcome and tumor progression in patients with OSCC (Liu *et al.*, 2018b; Wang *et al.*, 2008; Zhang *et al.*, 2016). A high number of M2 macrophages infiltrating the tumor is also associated with poor prognosis in OSCC. To evaluate the clinical significance of RACK1 and M2 macrophages, we analyzed 36 primary OSCC patient specimens by IHC staining (Table 1). The numbers of CD68^+^ macrophages (total macrophages, M0), CD11b^+^ macrophages (M1 macrophages) and CD206^+^ macrophages (M2 macrophages) were counted in 36 paraffin‐embedded tissues. High RACK1 protein expression was associated with more CD206^+^ macrophage cells; conversely, low RACK1 protein expression was associated with fewer CD206 positive macrophage cells (Fig. 1A). The expression of RACK1 correlated significantly with the M2 phenotype in OSCC (Fig. 1B, *r* = 0.698, *P* < 0.001). Survival analysis revealed that the number of CD68^+^ macrophage (M0) cells had no correlation with the OS of OSCC patients, consistent with the TCGA database analysis (Fig. S1A,B, *P* < 0.05). Notably, patients with a high M2/M1 ratio had a poorer OS than patients with a low M2/M1 ratio (Fig. 1C, *P* = 0.026), consistent with the TCGA database analysis (Fig. 1D, *P* < 0.01). These findings indicated that the ratio of M2/M1 macrophages affects the prognosis of OSCC but not the total amount of macrophages. RACK1 may affect the M2/M1 macrophage ratio in the OSCC microenvironment.

**Table 1:** Baseline characteristics of the patients with OSCC in our cohort.

Characteristic	Guangzhou cohort (*n* = 36)	*P*‐value*
	*n* (%)	
Age (mean ± SD)	61.56 ± 13.13	
< 60 years	14 (38.89)	0.191
≥ 60 years	22 (61.11)	
Sex		
Male	25 (69.44)	0.070
Female	11 (30.56)	
Smoking		
Never	19 (52.78)	0.778
Ever	17 (47.22)	
Drinking		
Never	23 (63.89)	0.439
Ever	13 (36.11)	
Differentiation		
High	29 (80.55)	0.246
Moderate	6 (16.67)	
Low	1 (2.78)	
Tumor stage		
T1	6 (16.67)	0.431
T2	16 (44.44)	
T3	8 (22.22)	
T4	6 (16.67)	
Nodal stage		
N0	17 (47.22)	0.355
N1–N3	19 (52.78)	
Clinical TNM stage		
I	4 (11.11)	0.858
II	10 (27.78)	
III	11 (30.56)	
IV	11 (30.56)	
Surgery type		
Local	2 (5.56)	0.669
Unilateral neck	29 (80.56)	
Bilateral neck	4 (11.11)	
Other	1 (2.78)	
Radiotherapy		
Yes	4 (11.11)	0.906
No	32 (88.89)	
Chemotherapy		
Yes	22 (61.11)	0.940
No	14 (38.89)	
Radiotherapy or chemotherapy		
Yes	24 (66.67)	0.962
No	12 (33.33)	

* *P*‐values of comparisons between studies were generated using a mixed linear model for continuous variables and Chi‐square test or Fisher's exact test for categorical variables.

### 3.2 RACK1 expression in OSCC cells inhibits macrophage recruitment *in vitro*

**Figure 2:**
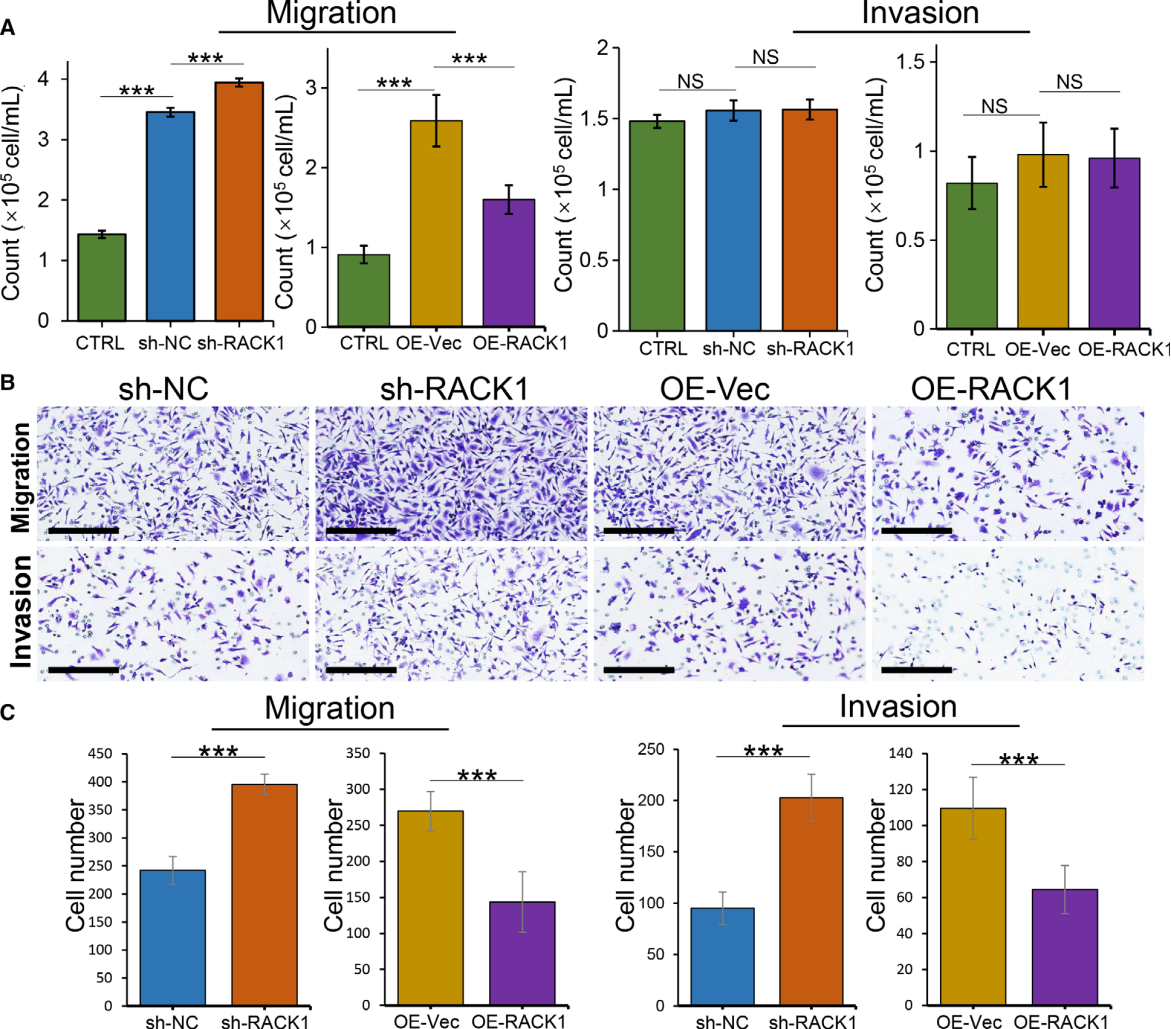
RACK1 inhibits the capacity of OSCC cells to recruit macrophages. (A) Transwell migration and Transwell Matrigel invasion assays used THP‐1 cells in the upper chamber and infected OSCC supernatants in the lower chamber. The average numbers of migrated and invaded cells were quantified (mean ± SD; ***P < 0.001). (B) Transwell migration and Transwell Matrigel invasion assays used RAW264.7 cells in the upper chamber and infected OSCC supernatants in the lower chamber (scale bars: 200 μm). (C) The average numbers of migrated and invaded RAW264.7 cells were quantified (mean ± SD; ***P < 0.001).

To uncover the biological function of RACK1 during macrophage recruitment in OSCC, both cell migration and invasion assays were performed. First, a stable HSC‐3 cell line with low RACK1 expression (sh‐RACK1) and a stable HSC‐4 cell line with RACK1 overexpression (OE‐RACK1) were generated as previously described (Zhang *et al.*, 2016). Then, we investigated the migration abilities of macrophages after coculture with conditioned media from OSCC cells with different levels of RACK1 expression. We found that RACK1 depletion promoted the ability of OSCC cells to induce THP‐1 cell migration (Fig. 2A, *P < *0.001). However, RACK1 expression did not affect the invasion of THP‐1 cells. Similarly, silencing RACK1 promoted the ability of OSCC cells to induce the migration and invasion of RAW264.7 cells (*P* < 0.001), whereas overexpressing RACK1 strongly induced inhibition (Fig. 2B,C). In addition, according to TCGA data, the mRNA expression of RACK1 is negatively correlated with the mRNA level of CD68, which is a biomarker for M0 (Fig. S1C, Spearman's rank correlation coefficient *r *= −0.1249, *P* = 0.0043). Collectively, these results indicate that RACK1 inhibits macrophage recruitment in the TME of OSCC.

### 3.3 RACK1 expression in OSCC inhibits macrophage activation but increases the proportion of M2 macrophages *in vitro* and *in vivo*

**Figure 3:**
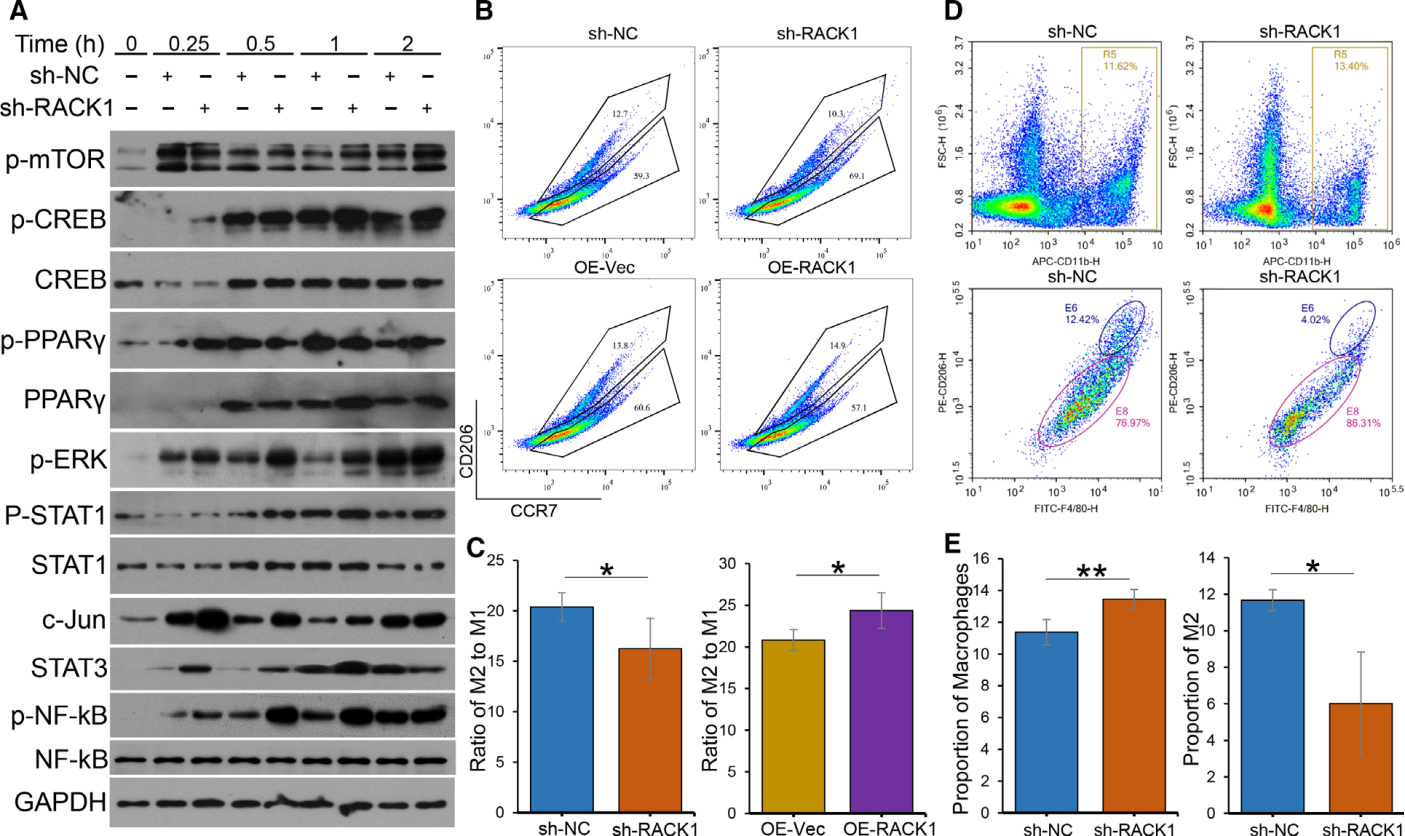
RACK1 inhibits macrophage activation but increases the proportion of M2 macrophages. (A) Immunoblots of p‐mTOR, CREB, p‐CREB, PPARγ, p‐PPARγ, p‐ERK, STAT1, p‐STAT1, c‐Jun, STAT3, NF‐κB and p‐NF‐κB in macrophages, induced from THP‐1 cells by 100 ng·mL^−1^ PMA for 24 h, cocultured with infected OSCC supernatants for the indicated times (0, 0.25, 0.5, 1 and 2 h). (B) Macrophages were induced from THP‐1 cells by PMA following incubation with transfected OSCC supernatants (sh‐NC, sh‐RACK1, OE‐Vec or OE‐RACK1) for 8 h. After centrifugation, the macrophages were stained with CD206 and CCR7 antibodies. The percentages and cell numbers of different macrophages were analyzed using flow cytometry. (C) Analysis of the M2/M1 ratio of different THP‐1‐induced groups detected by flow cytometry (mean ± SD; *P < 0.05). (D) Single cells were isolated from tumor tissues using collagenase IV and then stained with CD206, CD11b and F4/80 antibodies. The percentages and cell numbers of macrophages were analyzed using flow cytometry. R5: CD11b‐positive macrophages; E6: both CD206‐ and F4/80‐positive macrophages (M2). (E) Analysis of the numbers of M0 and M2 macrophages in tumor tissues from different groups detected by flow cytometry (mean ± SD; *P < 0.05, **P < 0.01).

Next, to determine the effect of RACK1 on macrophage activation in OSCC, we induced THP‐1 monocyte differentiation into macrophages by PMA treatment and then cocultured them with infected OSCC supernatants. The western blot results showed that M1‐ and M2‐related molecular pathways were activated in macrophages cocultured with OSCC supernatants. M2‐type macrophage pathway‐related molecules, such as mTOR and CREB, were not changed significantly, and PPARγ and STAT3 were significantly increased after coculture with RACK1‐silenced OSCC supernatant. Interestingly, levels of M1 macrophage pathway molecules, such as ERK, AP‐1 (AP‐1 consists of c‐Jun and c‐Fos) and NF‐κB, were dramatically higher than those in the control group after coculture with RACK1‐silenced OSCC supernatant; STAT1 levels, however, did not change (Fig. 3A). These results suggest that following the downregulation of RACK1 expression, both M1‐ and M2‐associated factors are significantly increased.

The flow cytometry results showed that RACK1 silencing inhibited (*P* = 0.038) whereas RACK1 overexpression increased (*P* = 0.022) the proportion of M2 macrophages *in vitro* (Fig. 3B,C). In addition, to investigate whether RACK1 contributes to macrophage recruitment in the tumorigenic environment, sh‐NC and sh‐RACK1 HSC‐3 cells were grafted into the flanks of nude mice by subcutaneous injection (Fig. S2). Then, the proportion of CD11b^+^ (a common marker of mouse macrophages) cells in the tumor tissues was assessed by flow cytometry. There were more CD11b^+^ (M0) cells in RACK1‐silenced tumors than in control tumors (Fig. 3D,E, *P* < 0.01). Interestingly, the RACK1‐silenced group had fewer CD11b^+^CD206^+^F4/80^+^ cells (M2) than the control group (Fig. 3D,E, *P* = 0.021). Combined, these results indicate that RACK1 inhibits macrophage activation but increases the proportion of M2 macrophages.

### 3.4 RACK1 expression in OSCC cells increases the intratumoral M2/M1 ratio in an NF‐κB axis‐dependent manner

**Figure 4:**
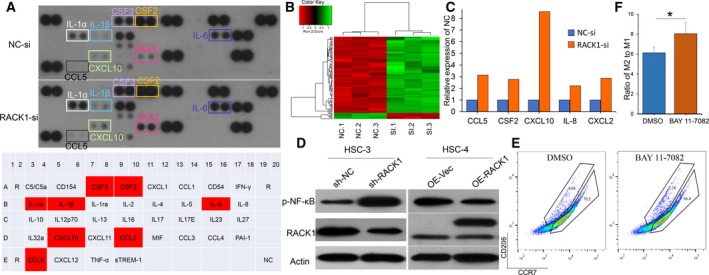
RACK1 increases the M2/M1 ratio in an NF‐κB axis‐dependent manner. (A) Human cytokine array analysis of the conditioned medium from HSC‐3 control cells and HSC‐3 cells with RACK1 silencing for 36 h. (B) Unsupervised hierarchical clustering analysis by complete linkage between si‐NC‐ and si‐RACK1‐transfected HSC‐3 cells for 36 h. (C) Analysis of CCL5, CSF2, CXCL10, IL‐8 and CXCL2 mRNA levels in HSC‐3 cells transfected with si‐NC‐ or si‐RACK1 for 36 h (*P* < 0.05). (D) Western blots for p‐NF‐κB and RACK1 in two infected OSCC cell lines. (E) Macrophages were induced from THP‐1 cells by PMA and cocultured with sh‐NC HSC‐3 cell supernatant (with DMSO or BAY 11‐7082 included) for 8 h. After centrifugation, the macrophages were stained with CD206 and CCR7 antibodies. The percentages and cell numbers of macrophages were analyzed using flow cytometry. (F) Analysis of the M2/M1 ratio in different induction groups (DMSO or BAY 11‐7082) (mean ± SD; *P < 0.05).

To investigate differences in the expression of cytokines in the conditioned medium from OSCC cells with or without RACK1 expression, human cytokine and gene arrays were performed. The levels of three cytokines, CCL5, IL‐6 and CSFs, were remarkably increased in RACK1‐ablated supernatant compared with those in the control group (Fig. 4A). Furthermore, the gene chip analysis also showed that the gene expression levels of CCL5 and CSFs were remarkably increased after RACK1 depletion (Fig. 4B,C, *P* < 0.05). TRRUST (http://www.grnpedia.org/trrust/) was used to analyze several related cytokines [RACK1, CCL2, CCL5, CSF2, CXCL10, CXCL2, IL‐1B, IL‐6 and tumor necrosis factor (TNF)], and we found that NF‐κB was the key protein in regulating these factors. These results were confirmed with transfected OSCC cells and 293 cells by western blot analysis. Compared with that in the control cells, p‐NF‐κB was reduced in the RACK1‐silenced cells, whereas it was increased in the RACK1‐overexpressing cells (Fig. 4D). To explore the effect of inhibiting NF‐κB activity, BAY 11‐7082 was added to conditioned media from HSC‐3 cells. As a result, BAY 11‐7082 significantly increased the proportion of M2 macrophages compared with DMSO (Fig. 4E,F, *P* = 0.018). These results suggest that RACK1 decreases IL‐6, CCL5 and CSF levels and increases the M2/M1 ratio in an NF‐κB axis‐dependent manner.

## 4 Discussion

Oral squamous cell carcinoma has an intricate TME that includes immune cells. Among these cells, TAM (M2) are believed to participate in the development and progression of OSCC (Fujii *et al.*, 2012; He *et al.*, 2014; Kubota *et al.*, 2017; Lei *et al.*, 2016; Matsuoka *et al.*, 2015; Sato‐Kaneko *et al.*, 2017). Current studies of macrophages in OSCC are not thorough and are limited to IHC staining experiments. There have been controversial conclusions about the relationship between TAM and the prognosis of OSCC patients (Alves *et al.*, 2018). Our study revealed the correlation between RACK1 and TAM and the underlying mechanisms through a clinical cohort analysis and *in vitro* and *in vivo* experiments. To exclude the influence of other immune cells as much as possible, especially for T cells, we used BALB/c nude mice, without mature T cells but with macrophages, to construct the animal model. Further investigation indicated that RACK1 could inhibit macrophage recruitment and increase the M2/M1 ratio in an NF‐κB axis‐dependent manner, thus promoting the development of OSCC.

The relationship between TAM and the prognosis of OSCC patients is controversial. Although there is substantial evidence that a larger number of CD68^+^ macrophages indicates a poorer prognosis (Liu *et al.*, 2008; Lu *et al.*, 2010; Ni *et al.*, 2015), some reports have suggested that there is no correlation (Costa *et al.*, 2013; Fang *et al.*, 2017; Marcus *et al.*, 2004). In addition, Udeabor *et al.* (2017) found that there were more M1 macrophages than M2 macrophages in most OSCC tissues, but the M2 number was higher than the M1 number in only 15% of the tissues. This result may be associated with both the antitumor and tumor‐suppressive properties of TAM, and the composition of TAM is different for different stages of OSCC. Unlike the controversial conclusions about the correlations between the total number of macrophages and the prognosis of OSCC, most studies have supported that more M2‐type macrophages indicate a poorer prognosis in OSCC patients (Balermpas *et al.*, 2014; Fujii *et al.*, 2012; He *et al.*, 2014; Hu *et al.*, 2016; Matsuoka *et al.*, 2015; Sakakura *et al.*, 2016; Wang *et al.*, 2014b). Here, we found that the proportion of M2/M1 macrophages, but not the total amount of macrophages, affects the prognosis of OSCC patients. The results based on the TCGA database are consistent with ours.

In the TME, the recruitment and polarization of macrophages play an indispensable role in the development of tumors (DeNardo and Ruffell, 2019). Previous studies have revealed that various cytokines, such as CCL2 (Eggert *et al.*, 2016; Garzia *et al.*, 2018; Hartwig *et al.*, 2017; Long *et al.*, 2016; Shen *et al.*, 2017; Tan *et al.*, 2018a; Tsai *et al.*, 2018), CCL3/4/5/7 (Coma *et al.*, 2006; Mineharu *et al.*, 2012; Yan *et al.*, 2011), CXCL12 (Mineharu *et al.*, 2012; Yan *et al.*, 2011), VEGF (Horwitz *et al.*, 2014) and PDGF (Dewar *et al.*, 2005; Yang *et al.*, 2016), have chemotactic effects on circulating monocytes. Accordingly, our research provided evidence that the expression of IL‐6, CCL5 and CSFs in OSCC cells and the secreted IL‐6 levels in cell supernatants were significantly higher in the RACK1‐silenced group than in the control group. In other words, RACK1 could inhibit the expression of IL‐6, CCL5 and CSF in OSCC cells and their secretion and then inhibit macrophage recruitment to the TME of OSCC. Furthermore, during the polarization process of macrophages, AP‐1, NF‐κB and STAT1 activation is required for M1 polarization, and mTOR, PPARγ/δ and STAT6 activation is critical for M2 polarization (Czimmerer *et al.*, 2018; Zhu *et al.*, 2015). Our study demonstrated that both M1 and M2 key activation factors, such as AP‐1, NF‐κB, STAT1 mTOR, and PPARγ, were significantly increased after RACK1 was silenced in OSCC cells. Moreover, we found that RACK1 could inhibit the recruitment of macrophages and then induce a ‘large bang’ of inflammatory factors, which could induce M0 cell polarization into M1 or M2 macrophages and increase the M2/M1 ratio. Thus, RACK1 could inhibit the recruitment of macrophages, increase the ratio of M2/M1, and create a chronic uncontrollable inflammatory environment, which could affect the development and metastasis of tumors.

In the TME, chronic and persistent inflammation without obvious clinical symptoms, also known as chronic smoldering inflammation, is the fundamental cause of the occurrence and development of tumors (Balkwill *et al.*, 2005). Here, we revealed that RACK1 can inhibit the activation of NF‐κB, consistent with previous results (Yao *et al.*, 2014). RACK1 can regulate the expression and secretion of proinflammatory cytokines and macrophage chemotactic factors in tumor cells, inhibit the massive recruitment of macrophages and severe inflammatory reactions, induce chronic smoldering inflammation in the TME and promote the progression of tumors, which may be an important reason why RACK1 promotes OSCC development. Considering the plasticity of TAM and the controversial relationship between the number of M0 and the prognosis of OSCC patients, targeting M2 macrophages instead of all macrophages may be a better treatment strategy.

The present study clearly has limitations that must be acknowledged. The RACK1 concentration and M2/M1 ratio are positively correlated, but the mechanism by which RACK1 promotes macrophage polarization to induce TAM to differentiate towards an M2‐like phenotype requires further investigation. Last but not least, whether RACK1 regulates NF‐κB directly remains to be explored.

## 5 Conclusions

**Figure 5:**
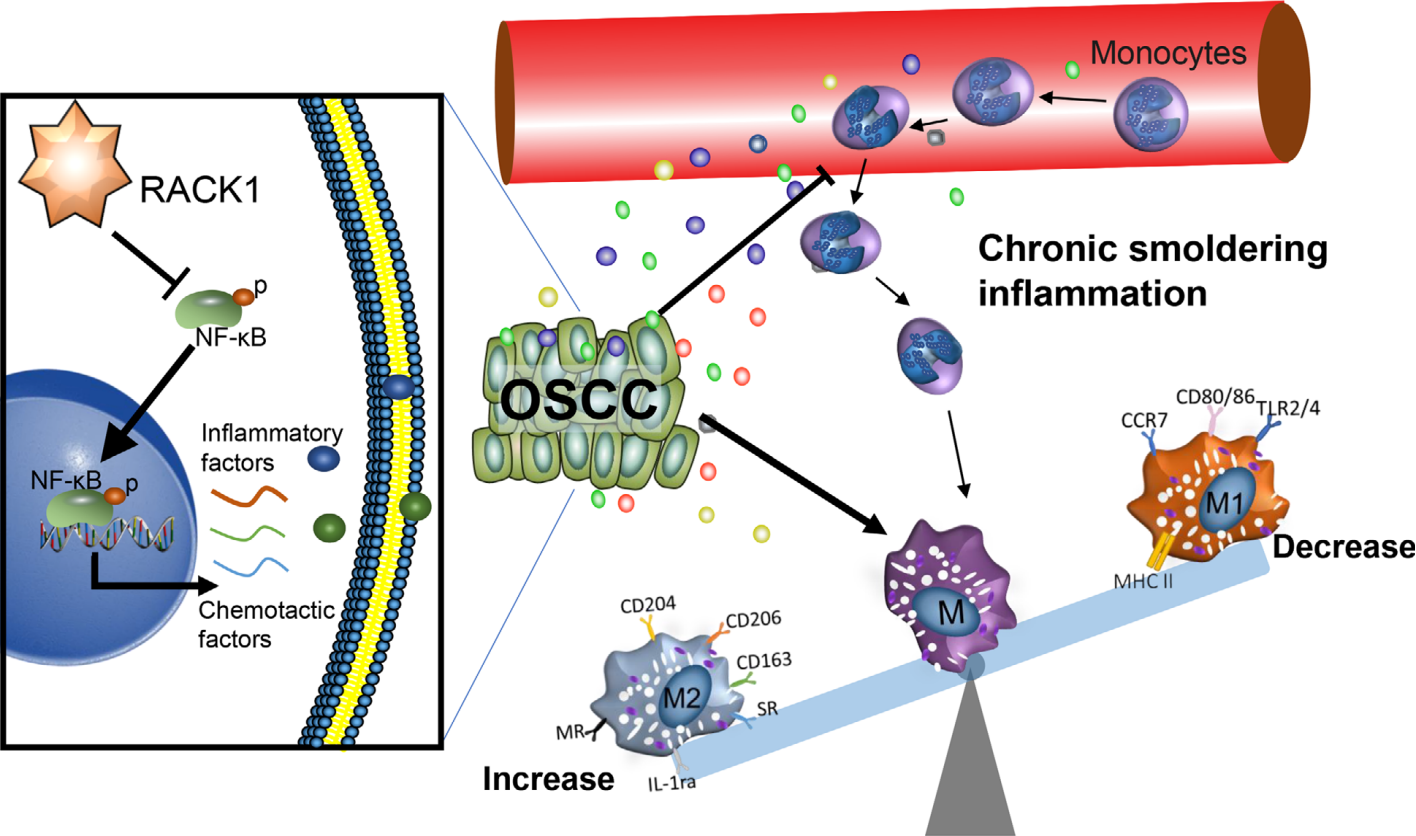
Schematic model by which RACK1 promotes the progression of OSCC. RACK1 inhibits the activation of NF‐κB, regulates the expression and secretion of proinflammatory factors and macrophage chemokines, inhibits the massive recruitment of macrophages and severe inflammatory reactions, induces a chronic smoldering inflammation microenvironment and promotes the development of tumors.

In conclusion, our study demonstrates that RACK1 and M2 macrophages are upregulated and are associated with a poor prognosis in OSCC through a clinical cohort analysis. Further investigation indicated that RACK1 could inhibit macrophage recruitment and increase the M2/M1 ratio in an NF‐κB axis‐dependent manner, thus promoting the development of OSCC (Fig. 5), suggesting that RACK1 could be used as a potential therapeutic target for antitumor immunity.

## Conflict of interest

The authors declare no conflicts of interest.

## Author contributions

HXD, SL and JJL conceived the study. SL, JJL, DJL, FYY, ZHW and JKW carried out the experiments. YZ, LJ, NJ, XZ, JL and QMC analyzed the data. HXD, SL and JJL wrote the manuscript. All authors read and approved the final version of the manuscript.

## Supporting information


**Fig. S1.** M0 number is not correlated with OSCC prognosis but is negatively associated with RACK1 at the mRNA level. (A) OS according to CD68 protein expression in a clinical cohort of OSCC patients (*n* = 37, *P* = 0.9829). (B) OS according to CD68 mRNA expression in OSCC TCGA data (*n* = 460, *P* = 0.5385). (C) Correlation between RACK1 and CD68 mRNA expression in the TCGA database (Spearman's rank correlation coefficient *r* = −0.1249, *P* < 0.01).

**Fig. S2.** The tumor volumes of the sh‐RACK1 group were smaller than those of the sh‐NC group.
