# Supplementary figures and images for: RACK1 promotes cancer progression by increasing the M2/M1 macrophage ratio via the NF‐κB pathway in oral squamous cell carcinoma
Source: Mol Oncol. 2020 Feb 20;14(4):795–807. doi: 10.1002/1878-0261.12644 (PMC7138402; doi:10.1002/1878-0261.12644)

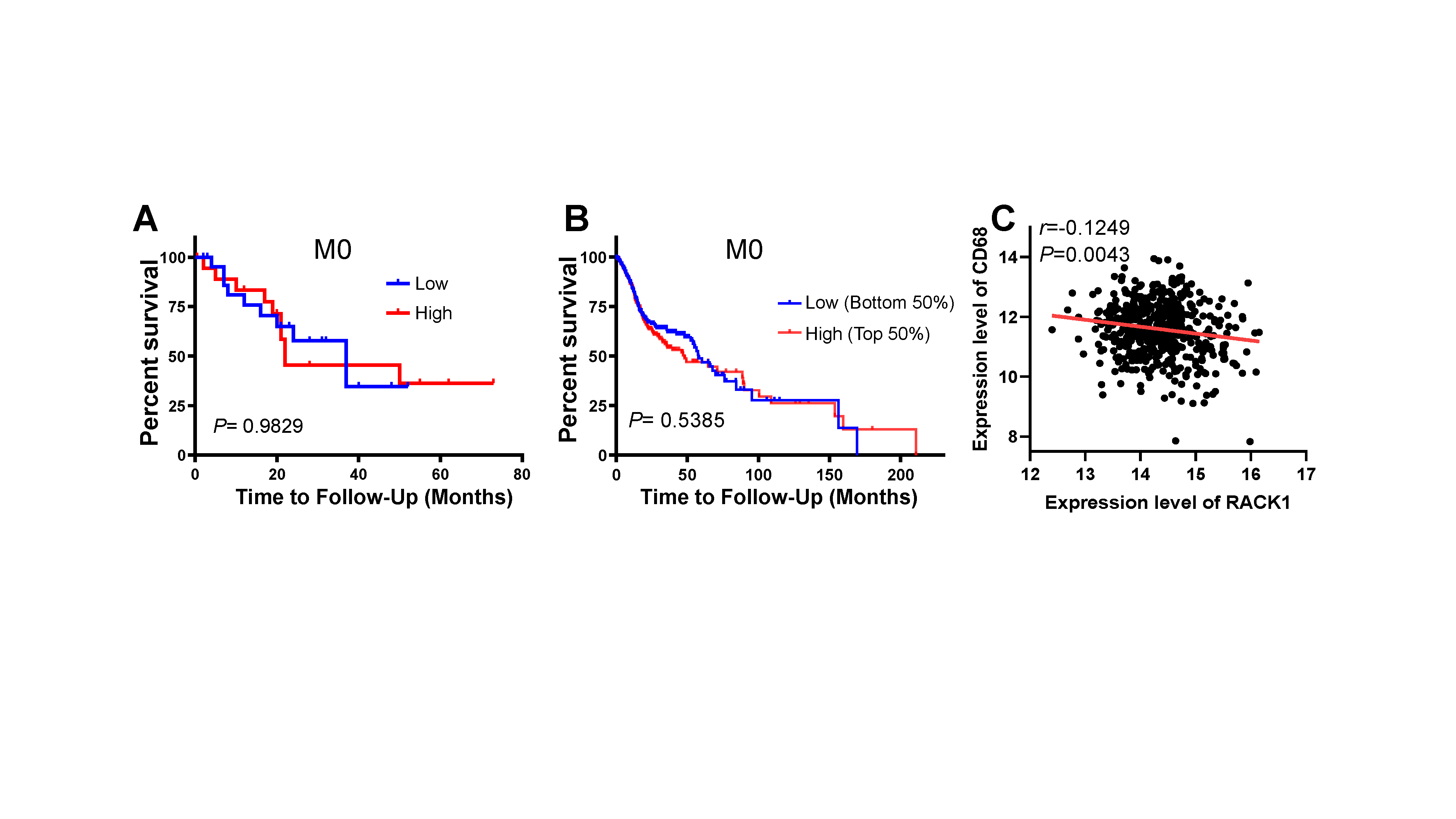

Supplement: Supplementary file 1 — Fig. S1. M0 number is not correlated with OSCC prognosis but is negatively associated with RACK1 at the mRNA level. (A) OS according to CD68 protein expression in a clinical cohort of OSCC patients (n = 37, P = 0.9829). (B) OS according to CD68 mRNA expression in OSCC TCGA data (n = 460, P = 0.5385). (C) Correlation between RACK1 and CD68 mRNA expression in the TCGA database (Spearman's rank correlation coefficient r = −0.1249, P < 0.01). [file MOL2-14-795-s001.tif]

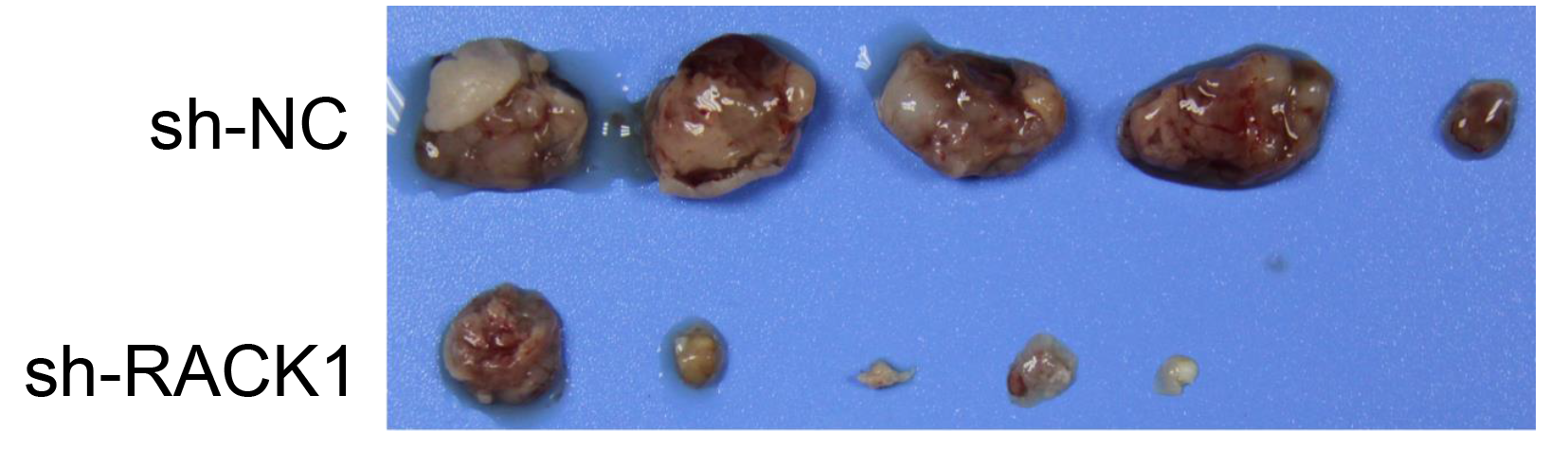

Supplement: Supplementary file 2 — Fig. S2. The tumor volumes of the sh‐RACK1 group were smaller than those of the sh‐NC group. [file MOL2-14-795-s002.tif]
